# Detection of Marker miRNAs, Associated with Prostate Cancer, in Plasma Using SOI-NW Biosensor in Direct and Inversion Modes

**DOI:** 10.3390/s19235248

**Published:** 2019-11-29

**Authors:** Yuri Ivanov, Tatyana Pleshakova, Kristina Malsagova, Leonid Kurbatov, Vladimir Popov, Alexander Glukhov, Alexander Smirnov, Dmitry Enikeev, Natalia Potoldykova, Boris Alekseev, Daniyar Dolotkazin, Andrey Kaprin, Vadim Ziborov, Oleg Petrov, Alexander Archakov

**Affiliations:** 1 Institute of Biomedical Chemistry (IBMC), Moscow 119121, Russia; yurii.ivanov@rambler.ru (Y.I.); t.pleshakova1@gmail.com (T.P.); leonid15@mail.ru (L.K.); alexander.archakov@ibmc.msk.ru (A.A.); 2 Joint Institute for High Temperatures of Russian Academy of Sciences, Moscow 125412, Russia; ziborov.vs@yandex.ru (V.Z.);; 3 Rzhanov Institute of Semiconductor Physics, Siberian Branch of Russian Academy of Sciences, Novosibirsk 630090, Russia; popov@isp.nsc.ru; 4 Joint-Stock Company “Novosibirsk Plant of Semiconductor Devices & DC”, Novosibirsk 630082, Russia; gluhov@nzpp.ru; 5 Russian Union of Industrialists and Entrepreneurs, Moscow 109240, Russia; arhsmirnov@mail.ru; 6 Institute for Urology and Reproductive Health, Sechenov University, Moscow 119992, Russia; enikeev_dv@mail.ru (D.E.); natalis8282@mail.ru (N.P.); 7 Federal State Budgetary Institution National Medical Research Radiological Center of the Ministry of Health of the Russian Federation, Moscow 125284, Russia; byalekseev@mail.ru (B.A.); daniyar.dolotkazin@gmail.com (D.D.); kaprin@mail.ru (A.K.)

**Keywords:** prostate cancer, miRNA, silicon-on-insulator, nanowire biosensor

## Abstract

Information about the characteristics of measuring chips according to their storage conditions is of great importance for clinical diagnosis. In our present work, we have studied the capability of chips to detect nanowire biosensors when they are either freshly prepared or have been stored for either one or two years in a clean room. Potential to detect DNA oligonucleotides (oDNAs)—synthetic analogues of microRNAs (miRNAs) 198 and 429 that are associated with the development of prostate cancer (PCa)—in buffer solution was demonstrated using a nanowire biosensor based on silicon-on-insulator structures (SOI-NW biosensor). To provide biospecific detection, nanowire surfaces were sensitized with oligonucleotide probes (oDNA probes) complimentary to the known sequences of miRNA 183 and 484. In this study it is demonstrated that freshly prepared SOI-NW biosensor chips with n-type conductance and immobilized oDNA probes exhibit responses to the addition of complimentary oDNAs in buffer, leading to decreases in chips’ conductance at a concentration of 3.3 × 10^−16^ M. The influence of storage time on the characteristics of SOI-NW biosensor chips is also studied herein. It is shown that a two-year storage of the chips leads to significant changes in their characteristics, resulting in “inverse” sensitivity toward negatively charged oDNA probes (i.e., through an increase in chips’ conductance). It is concluded that the surface layer makes the main contribution to conductance of the biosensor chip. Our results indicate that the detection of target nucleic acid molecules can be carried out with high sensitivity using sensor chips after long-term storage, but that changes in their surface properties, which lead to inversed detection signals, must be taken into account. Examples of the applications of such chips for the detection of cancer-associated microRNAs in plasma samples of patients with diagnosed prostate cancer are given. The results obtained herein are useful for the development of highly sensitive nanowire-based diagnostic systems for the revelation of (prostate) cancer-associated microRNAs in human plasma.

## 1. Introduction

Prostate cancer (PCa) is a malignant tumor of the prostate, whose etiology and pathogenesis are still understudied. PCa is among the most common types of malignant tumors (according to the World Health Organization (WHO)). According to the statistics, prostate cancer has been ranked second after lung cancer among other malignant tumors in men across Russia over the past decade. The incidence rate of prostate cancer in Russia has tripled from 19.01 to 57.22 cases per 100,000 people over the past 15 years from 2001 to 2015 (according to the National Medical Research Radiological Center of the Ministry of Health of the Russian Federation).

Such a difficult situation is mainly associated with late diagnosis of the disease [1]. Currently available methods of instrumental examination and associated diagnostic procedures are far from perfect. The main screening methods include clinical examination and biopsy. The application of magnetic resonance imaging (MRI) is becoming more popular [2]. The biopsy method allows the determination of the types of cells that are susceptible to proliferation, detecting histological features, genetic damage and other problems associated with oncology [3]. Biopsy study, on the other hand, is limited to an indeterminate interpretation of the results associated with various individual structural features of the mammary gland; in addition, it is an invasive method that causes patient discomfort and remains ineffective at the early stage of the disease [1]. Therefore, the development of novel methods for PCa diagnosis at an early stage is an important field of research.

Potential next-generation methods include those based on nanowire detection, which allows for the registration of protein markers in biological fluid at low concentrations (<10^−13^ M) when the pathological process is at an early stage of development [4]. In addition to high sensitivity, this type of detection allows for label-free real-time analysis.

The SOI-NW biosensor is a biosensor based on silicon-on-insulator (SOI) structures. SOI nanowire structures are formed with the use of complementary metal-oxide-semiconductor (CMOS)-compatible technology by gas phase reduction and lithography [5]. This biosensor has been found to be an efficient tool for the detection of proteins and nucleic acids with extremely high sensitivity at levels from 10^−17^ to 10^−14^ M [6,7,8].

MicroRNAs have recently been used as biomolecular markers of early stages of cancer [9,10,11]. Gao A. et al., demonstrated the possibility for the specific revelation of lung cancer by NW-biosensor detection of marker miRNA-126 at a concentration of 10^−16^ M [12]. The literature contains discussions on the following miRNAs as potential markers: miR1246, miR-4634, miR1307-3p, miR-6875-5p, miR-6861-5p, and (miR)-10b [13,14].

Our present research has demonstrated the possibility to detect DNA oligonucleotides (oDNAs)—the synthetic analogues of PCa-associated miRNAs—in buffer solution, using SOI-NW biosensor chips. The surface of the sensor structures was sensitized with immobilized molecular probes in the form of DNA oligonucleotides complimentary to the target oDNAs. It has been shown that the SOI-NW biosensor can be used to effectively detect DNA oligonucleotides in buffer solution with a high concentration sensitivity of ~10^−16^ M.

The effect of storage time on the characteristics of SOI-NW biosensor chips made with the use of CMOS-compatible technology was also studied. The findings have shown that a two-year storage time leads to a significant change in their characteristics, resulting in an “inversed” sensitivity towards registration of negatively charged oDNA probes. Taking into account that it is the change in the surface layer that leads to inverse conductance during a two-year storage of the chip, it is concluded that the surface layer of the chip makes a significant contribution to the modulation of its conductance and its sensitivity toward miRNA. The results obtained herein indicate that the detection of target nucleic acid molecules can be carried out with high sensitivity using sensor chips after long-term storage, but that changes in their surface properties leading to inversed detection signal must be taken into account. The possibility of detecting microRNAs isolated from plasma of prostate cancer patients is also demonstrated herein. Thus, our results must be taken into account in the development of highly sensitive nanowire-based diagnostic systems for the revelation of PCa-associated microRNAs in human plasma.

## 2. Materials and Methods

### 2.1. Chemicals and Oligonucleotides

*Chemicals*

The cross-linker, 3,3′-dithiobis (sulfosuccinimidyl propionate) (DTSSP) was purchased from Pierce (USA); potassium phosphate monobasic (KH_2_PO_4_), dimethyl sulfoxide (DMSO), and 3-aminopropyltriethoxysilane (APTES) were purchased from Sigma Aldrich (St. Louis, MA, USA). Methanol (CH_3_OH) was from Sigma (St. Louis, MA, USA). Hydrofluoric acid (HF) were from Reakhim (Moscow, Russia). Deionized water was obtained in a Simplicity UV purification system (Millipore, Molsheim, France).

*Oligonucleotides*

The following oDNA probes purchased from Evrogen, Russia were used for modification of the SOI-NW surface (Table 1).

In accordance with their numbering, these oDNA probes were complimentary to DNA oligonucleotides, which were used as target molecules in the model system analysis as synthetic analogues of the sequences published in [15]. The sequences of oDNA and miRNA are shown below (Table 2). The DNA oligonucleotide, used as the control probe, was non-complimentary to both cs_1 nor and cs_2 and had the following sequence: (NH_2_)-(T) _10_-GAGGCTGAGATGTTGCTACTGCTATGAGAAGATATGTCAAGCCAGAGTAT («control»).

### 2.2. SOI-NW Sensors Fabrication

The design and characteristics of the SOI-NW structures used as sensor elements are described in [5,6,8]. SOI-NW structures with n-type conductance were used. These structures were freshly prepared and stored for either one or two years in clean room conditions. The thickness of the silicon cut-off layer was 32 nm, and the buried oxide (BOX) thickness was 300 nm. In the experiments, the width of the sensor elements was 3 nm, the thickness was 32 nm, the length was 10 nm, and the number of SOI-NWs on the chip was 12. The SOI-NW biosensor scheme is described in [5,8].

This study design used a 500-mL measuring cell, the bottom of which was the chip sensitive area with the SOI-NW structures. The diameter of the sensitive area was about 2 mm. The solution stirring was carried out with a stirrer at 3000 rpm.

### 2.3. Sensor Surface Modification

The surfaces of chips (either freshly prepared or stored for one or two years) were treated according to the standard procedure using isopropanol, water, and the solution of HF in CH_3_OH to remove organic contaminants by sequential double treatment [5,6,8]. The freshly prepared chips were then additionally placed into the ozonizer to form hydroxyl groups on the surface of the SOI-NW structures [5]. The chips’ surfaces were silanized in APTES vapors according to the protocol described elsewhere [5,8].

In cases of intensive oxide layer removal from the surfaces of chips stored for more than two years, it was necessary to remove structures formed during long-term storage, and chip surfaces were treated with isopropanol, water, and a five-time treatment of 1:50 *v*/*v* HF in CH_3_OH.

### 2.4. Covalent Immobilization of Oligonucleotide Probes

The oDNA probes (probe_1, probe_3, «control») were covalently immobilized on the modified surface of the SOI-NW with the use of the DTSSP cross-linker [6]. For this purpose, solutions (V = 3 nL) containing one of the types of oligonucleotide probe (1 μM) in potassium phosphate buffer (50 mM, pH 7.4) were precisely applied onto the surface of each individual SOI-NW. Solutions were applied using the Piezorray non-contact dispensing system (PerkinElmer, Inc., Waltham, MA, USA). Solutions were incubated for 30 min at 15 °C and 80% humidity on the surface of the SOI-NW structures. After that, the sensor chip area was washed in deionized water.

### 2.5. Preparation of Solutions of TARGEt oDNAs in Buffer

Target oDNA solutions with concentrations ranging from 3.3 × 10^−12^ M to 3.3 × 10^−18^ M were prepared from the initial solution (100 μM in 50 mM potassium phosphate buffer, pH 7.4) with the use of 10-fold serial dilution in a working buffer solution (1 mM potassium phosphate buffer, pH 7.4). The solution was kept in a shaker for 30 min at 10 °C at each stage of dilution. All the solutions were prepared immediately before measurements.

### 2.6. Electrical Measurements

We carried out all the electrical measurements with the use of an analog-to-digital converter, while the support of the SOI structures was used as a control electrode (transistor gate). The registration of the drain-source *I_ds_* dependence on the value of the voltage supplied to the gate *V_g_* so as the volt-ampere characteristics were obtained at *V_g_* from 0 to 60 V and *V_ds_* = 0.1 V. The registration of the drain-source current *I_ds_* dependencies on time *(t)* were carried out at gate voltage *V_g_* = 55 V and at *V_ds_* = 0.1 V.

### 2.7. NW Biosensor Measurements

The analyzed solution (150 μL, in 1 mM potassium phosphate buffer) containing one of the types of oDNA (“cs”) at a concentrations from 3.3 × 10^−12^ M to 3.3 × 10^−18^ M was added into the measuring cell containing 300 μL of buffer solution (1 mM potassium phosphate buffer). Thus, the initial concentration of the solution decreased threefold. Control experiments were performed under similar conditions, but the oDNA-free buffer was added.

SOI-NW biosensor signals were recorded in real time. To account for the nonspecific sorption of the target objects, chips containing working sensors also contained a pair of control sensors, the surfaces of which were sensitized by the “control” probe. The obtained results are presented in the form of Δ*I_ds_(t)* dependencies reflecting the differential signal between the working (with immobilized probe_1 or probe_3 oligonucleotides) and the control SOI-NW sensor. The detection of DNA oligonucleotides and miRNA were performed in buffer with a low salt concentration (1 mM potassium phosphate buffer) in order to exclude the effects of Debye screening [16].

Our experiments concerned the effects of storage time and clean room conditions on the performance of SOI-NW sensor chips under the following conditions: (1) freshly prepared chips; (2) chips stored for one year after fabrication; (3) chips stored for two years after fabrication; and (4) treating the chip that was stored for two years after fabrication five times with HF to intensively remove the surface layer.

### 2.8. Plasma Samples

In our research, plasma samples were obtained from patients diagnosed with prostate cancer. The patients were examined in Institute of Urology and Reproductive Health (Sechenov University) and in the National Medical Research Radiological Center of the Ministry of Health of the Russian Federation (Moscow, Russia). In control experiments, we used plasma samples from a healthy volunteer. Plasma samples nos. 2, 36, and 44 (see Table 3) were tested. All experiments using serum were performed in compliance with order no. 1177n (Ministry of Health of Russian Federation, 20 December 2012). Plasma samples were collected from patients according to the patient examination protocol. This study was approved by independent ethical committees organized on the basis of the organizations that provided the samples. Written informed consent was obtained from the patients and from healthy volunteer authorizing their participation in the study and the use of the biological material. All samples were deactivated prior to their use in the study to provide biological safety.

The blood samples were taken before treatment on an empty stomach using the cubital vein vacutainers with a 3.8% Na citrate anticoagulant (S-Monovett, SARSTEDT, Nümbrecht, Germany) then centrifuged at 3000 rpm for 6 min at RT. Each plasma sample (500 μL) was collected in two dry test tubes, frozen, and stored at −70 °C prior to the analysis.

To extract miRNAs from the blood plasma samples, miRCURY RNA Isolation Kit Biofluids were used.

## 3. Results

### 3.1. Results of Biosensor Measurements with Use of a Freshly Prepared Chip

#### 3.1.1. Current–Voltage Characteristics of a Freshly Prepared Chip with Reference to the SOI-NW Biosensor

**Figure 1:**
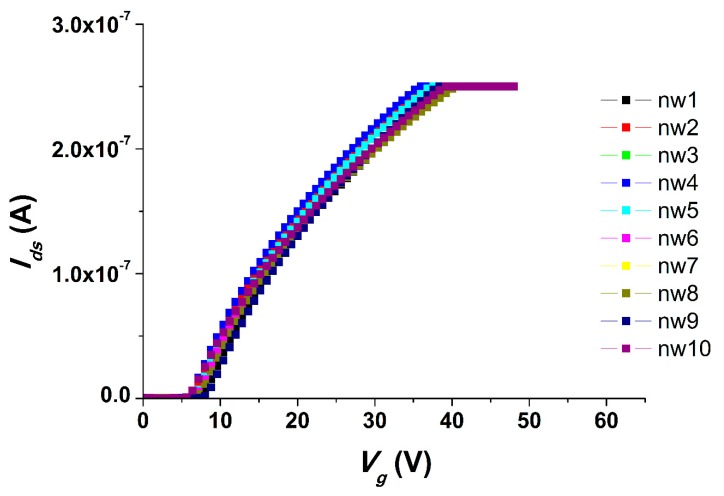
Typical current–voltage characteristics (CVCs) obtained for the freshly prepared chip. Experimental conditions: 1 mM potassium phosphate buffer, *V_g_* from 0 to 60 V, *V_d_*_s_ = 0.1 V, solution volume in the cell: 300 µL; chip with 3-µm-wide silicon-on-insulator nanowires (SOI-NWs) of n-type conductance.

Current–voltage characteristics (CVCs) were measured in order to determine the working capacity of the chip. Figure 1 displays the CVCs obtained for a freshly prepared chip in a buffer solution.

Figure 1 shows that the operating values of the CVCs of the chip are observed when a positive voltage of 6–7 V is applied to the gate. Thus, the chip exhibited the anticipated CVCs for an n-type field effect transistor.

#### 3.1.2. Biospecific Detection of DNA Oligonucleotides in Buffer Solution with a Freshly Prepared Chip

**Figure 2:**
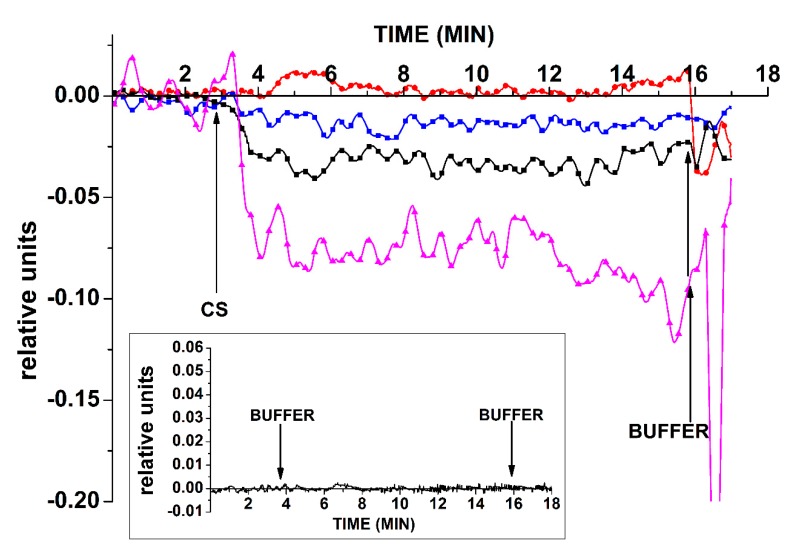
Typical sensograms obtained upon cs_1 oDNA detection with the freshly prepared chip. Experimental conditions: 1 mM potassium phosphate buffer, *V_g_ =* 55 V, *V_ds_* = 0.1 V, solution volume in the cell: 450 μL; the SOI-NW structure is sensitized with the oligonucleotide probe_1. The final concentration of cs_1 solutions in the cell: 3.3 × 10^−17^ M (red), 3.3 × 10^−16^ M (blue), 3.3 × 10^−15^ M (black), and 3.3 × 10^−14^ M (purple). The inset shows the results of the control experiments. Arrows indicate the addition of the analyzed solution and washing potassium phosphate buffer into the measuring cell.

For the oDNA-containing system, it has been shown that cs_1 samples can be detected in solution with SOI-NW sensor chips sensitized with oDNA probe_1. Figure 2 displays typical sensogram curves obtained before and after addition of the test solutions of the cs_1 DNA oligonucleotide into the measuring cell to a concentration from 0 to 3.3 × 10^−14^ M. In control experiments, when buffer with *C_oDNA_ =* 0 was added into the measuring cell containing buffer solution, no change in the biosensor signal was registered. Also, no change in the signal was registered upon analysis of solutions with *C_oDNA_ =* 10^−18^ M and *C_oDNA_ =* 10^−17^ M. We can also see that the biosensor signal magnitude decreased with a decrease in the concentration of the added oDNA from 3.3 × 10^−14^ M to 3.3 × 10^−16^ M. Figure 2 demonstrates that, when the cs_1 solution was added into the cell, the expected decrease in the signal from the SOI-NW with the immobilized probe_1 (which is complimentary to cs_1) was observed. The decrease in SOI-NW conductance was due to the adsorption of negatively charged molecules on its surface.

No change in the biosensor signal was observed by further increasing *C_oDNA_* to 3.3 × 10^−13^ and 3.3 × 10^−12^ M, which could be connected to the fact that a concentration saturation limit was reached, and this saturation effect could be the result of the high sensitivity of the biosensor. In our present study, oligonucleotide probes with lengths of 84–110 bp were immobilized onto the sensor surface. Upon hybridization of the probes with complimentary oDNA, a double-helix forms, whose diameter is known to be around *D* = 2 nm [17]. Thus, by assuming that all the oDNA molecules added into the measuring cell can be captured onto the nanowire sensor surface and hybridize on this surface, one can theoretically estimate the maximum number of oDNA molecules (*N_m_*) that can be captured on the NW sensor using the area (*S_nw_*) applied in our study: *S_nw_ = l* × *w* = 10 × 3 = 30 μm^2^ (30 × 10^6^ nm^2^). This number makes up $N_{m}=\frac{Snw}{Sm}=\;\frac{30*10^{6}}{\left(\frac{\pi }{4}\right)*2^{2}}$ = 10^7^ molecules, where *s_m_* is the cross-section area of the DNA double-helix formed upon the biospecific interation of the probe with the target. Taking into account that a volume of 450 µL contains 3 × 10^6^ molecules at an oDNA concentration of 3.3 × 10^−14^ M, a greater oDNA concentration of 3.3 × 10^−13^ M at the same volume will contain a greater number of molecules (3 × 10^7^) that exceeds the number that can be adsorbed onto the NW sensor. Therefore, it is possible that at a concentration of 3.3 × 10^−14^ M oDNA the saturation of the biosensor signal can be observed. Such an estimation is rather rough, and the number of oDNA molecules in the solution can be registered at a greater concentration, but this depends on the results of a specific procedure of sensitization of the NW sensor surface—specifically, it depends on the number of sensor-immobilized oligonucleotide probes and on the sensor chip construction.

**Figure 3:**
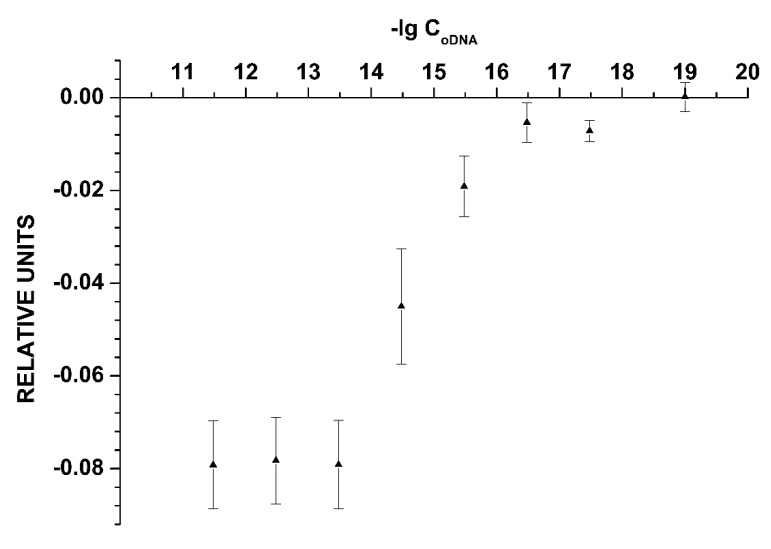
The dependence of the NW biosensor signal on the oDNA concentration in the analyzed solution obtained for the freshly prepared chip. Experimental conditions: 1 mM potassium phosphate buffer, *V_g_ =* 55 V, *V_ds_* = 0.1 V, solution volume in the cell: 450 μL; the SOI-NW structure is sensitized with the oligonucleotide probe_1. The final concentration of cs_1 solutions in the cell is from 3.3 × 10 ^−18^ M to 3.3 × 10^−12^ M. The number of technical replicates is *n* = 3.

Figure 3 shows the dependence of the NW biosensor signal on the oDNA concentration in the analyzed solution. This dependence is plotted on the basis of all data obtained in experiments with the freshly prepared sensor chip.

As seen in Figure 3, the minimum detectable concentration of the cs_1 oDNA was 3.3 × 10^−16^ M, since no significant change in the signal is observed at lower concentrations.

In the solutions containing oDNA cs_3 for the SOI-NW, sensitized probe_1, and the control, no signal other than noise was detected. As for the control experiments, when a working buffer (that did not contain miRNA) was added to the cell, either no response from the SOI-NW was observed or it amounted only to 1–2% of the current level (Figure 2, inset). This allows us to conclude that there was a biospecific interaction between molecular probes immobilized on the surface of the SOI-NW sensors and the target molecules from the analyzed solution.

### 3.2. Results of Biosensor Measurements with Use of a Chip Stored for One Year

#### 3.2.1. Current–Voltage Characteristics of a Chip after One Year of Storage with Reference to the SOI-NW Biosensor

**Figure 4:**
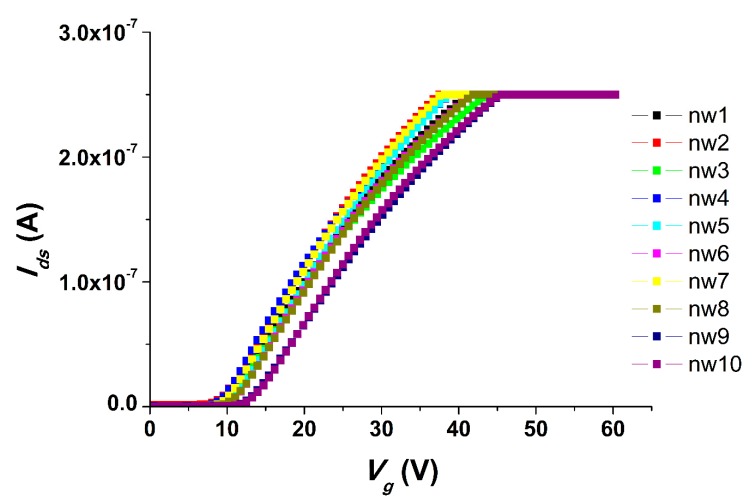
Typical current–voltage characteristics obtained for the chip after one year of storage. Experimental conditions: 1 mM potassium phosphate buffer, *V_g_* from 0 to 60 V, *V_d_*_s_ = 0.1 V, solution volume in the cell: 300 μL; chip with 3-µm-wide SOI-NWs of n-type conductance.

Current–Voltage characteristics (CVCs) were measured in order to determine the working capacity of the chip after one year of storage. Figure 4 displays the CVCs obtained for a freshly prepared chip in a buffer solution.

As seen from Figure 4, the chip exhibited “opening” (i.e. the current started to increase) upon application of a positive voltage at levels from 7 to 10 V to the gate.

#### 3.2.2. Biospecific Detection of DNA Oligonucleotides in the Buffer Solution with a Chip after One Year of Storage

**Figure 5:**
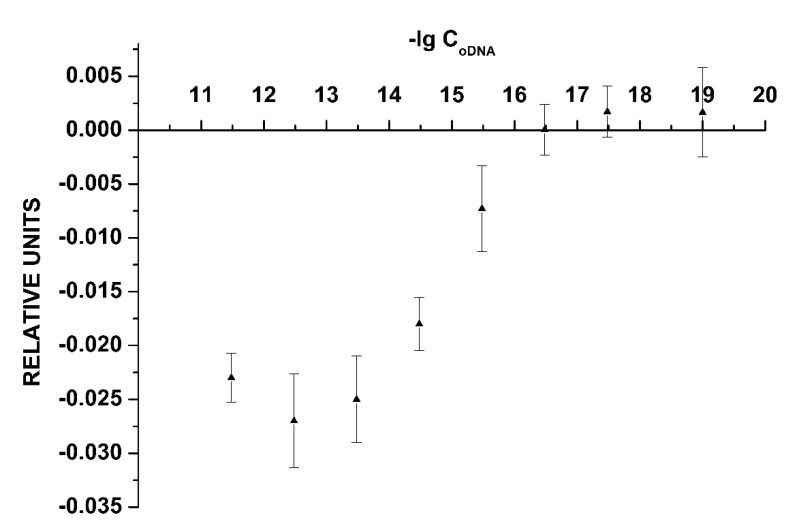
The dependence of the NW biosensor signal on the oDNA concentration in the analyzed solution obtained for the chip stored for one year. Experimental conditions: chip after one year of storage, 1 mM potassium phosphate buffer, *V_g_ =* 55 V, *V_ds_* = 0.1 V, solution volume in the cell: 450 µL; the SOI-NW structure is sensitized with the oligonucleotide probe_1. The final concentration of cs_1 solution in the cell is from 3.3 × 10^−18^ M to 3.3 × 10^−12^ M. The number of technical replicates is *n* = 3.

For the chip stored for one year, we observed a decrease in the sensors’ conductance upon addition of 3.3 × 10^−18^ to 3.3 × 10^−12^ M oDNA into the measuring cell, which is similar to the case with the freshly prepared chip. Figure 5 displays the dependence of the NW biosensor signal on the oDNA concentration in the analyzed solution. This dependence is plotted on the basis of all data obtained in experiments with the chip stored for one year.

Thus, one can conclude that the chip after one year of storage was able to perform oDNA detection with a high concentration sensitivity of ~3.3 × 10^−16^ M, which is comparable with that of the freshly prepared one.

### 3.3. Results of Biosensor Measurements with Use of a Chip Stored for TwoYyears

#### 3.3.1. Current–Voltage Characteristics of a Chip after Two Years of Storage with Reference to the SOI-NW Biosensor

**Figure 6:**
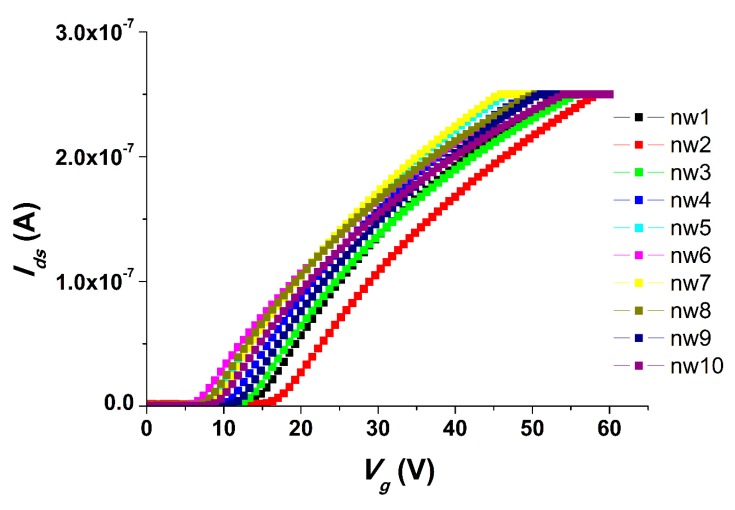
Typical current–voltage characteristics obtained for the chip after two years of storage. Experimental conditions: 1 mM potassium phosphate buffer, *V_g_* from 0 to 60 V, *V_ds_* = 0.1 V, solution volume in the cell: 300 μL; chip with 3-µm-wide SOI-NWs of n-type conductance.

While studying the effect of storage time on the chip characteristics, we conducted experimental work on the chip with two years of storage. Figure 6 shows the CVC of the chip in buffer solution.

This figure shows that the CVCs obtained for the chip stored for two years were similar to those for a freshly prepared chip. In other words, the operating CVC values of the chip could be observed while a positive voltage of 6–15 V was applied to the gate.

#### 3.3.2. Biospecific Detection of DNA Oligonucleotides in the Buffer Solution with the Use of a Two-Year-Old Chip

**Figure 7:**
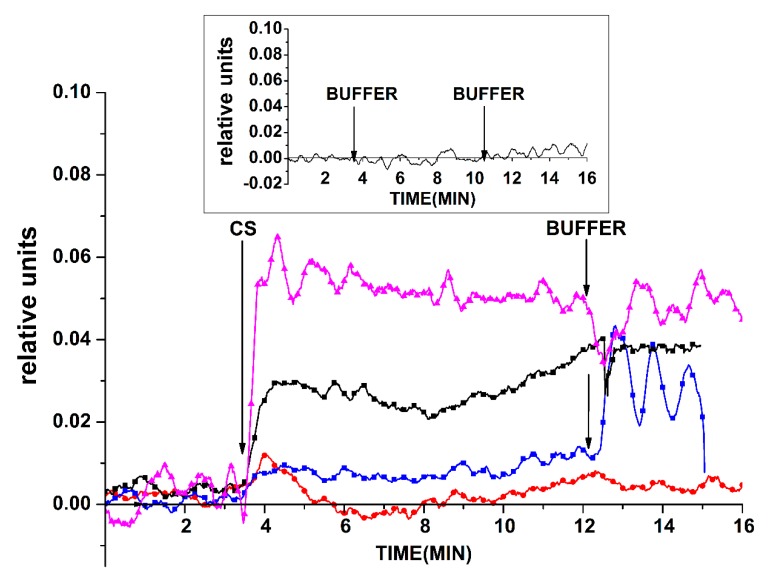
Typical sensograms obtained upon oDNA detection (cs_3) with the chip after two years of storage. Experimental conditions: 1 mM potassium phosphate buffer, *V_g_* = 55 V, *V_ds_* = 0.1 V, solution volume in the cell: 450 µL; the SOI-NW structure is sensitized with the oligonucleotide probe_3. The final concentration of cs_3 solutions in the cell is 3.3 × 10^−17^ M (red), 3.3 × 10^−16^ M (blue), 3.3 × 10^−15^ M (black), and 3.3 × 10^−14^ M (purple). The inset shows the results of the control experiments. Arrows indicate the addition of the analyzed solution and washing potassium phosphate buffer into the measuring cell.

Registration of the DNA oligonucleotide cs_3 was carried out in solution with the use of the SOI-NW sensitized with oDNA probe_3. Figure 7 shows the examples of time dependencies of the current recorded before and after adding the analyzed solutions with cs_3 to the SOI-NW biosensor cell in the concentration range from 10^−14^ to 10^−17^ M. As you can see from Figure 7, we were able to observe an unexpected increase in the signal from the SOI-NW immobilized by probe_3 while adding the analyzed solution with cs_3 to the cell, but not the expected decrease in the signal. The increase in the SOI-NW conductance was due to signal inversion that resulted in changes in the oxide properties on the surface of the SOI-NW structures. It is also obvious that the biosensor signal magnitude decreased with a decrease in the concentration of the added oDNA from 3.3 × 10^−14^ M to 3.3 × 10^−17^ M. The minimum detectable concentration of cs_3 oDNA was 3.3 × 10^−16^ M.

When a working buffer without target molecules was added into the cell in the control experiments, no response from the SOI-NW was observed, or it was insignificant (Figure 7, inset). This allows us to conclude that there was a biospecific interaction between molecular probes immobilized on the surface of the SOI-NW and the target molecules from the analyzed solution.

**Figure 8:**
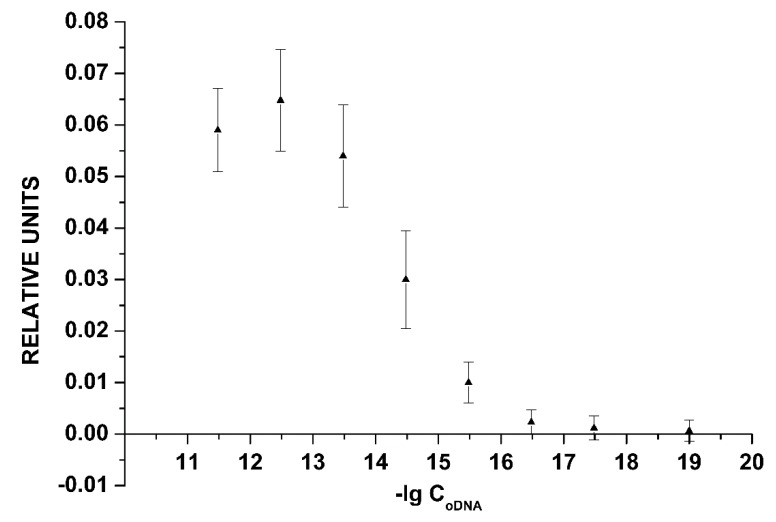
The dependence of the NW biosensor signal on the oDNA concentration in the analyzed solution obtained for the chip stored for two years. Experimental conditions: 1 mM potassium phosphate buffer, *V_g_ =* 55 V, *V_ds_* = 0.1 V, solution volume in the cell: 450 µL; the SOI-NW structure is sensitized with the oligonucleotide probe_3. The final concentration of cs_1 solutions in the cell is from 3.3 × 10^−18^ M to 3.3 × 10^−12^ M. The number of technical replicates is *n* = 3.

Figure 8 displays the dependence of the NW biosensor signal on the concentration of oDNA in the analyzed solution. This dependence is plotted on the basis of all data obtained in the experiments with the chip stored for two years.

As seen from Figure 7 and Figure 8, the minimum detectable concentration of the cs_3 oDNA was 3.3 × 10^−16^ M, as no significant change in the signal was observed at lower concentrations.

#### 3.3.3. DNA Detection Using a Two-Year-Old Chip after Intensive Removal of the Surface Layer from the Chip

**Figure 9:**
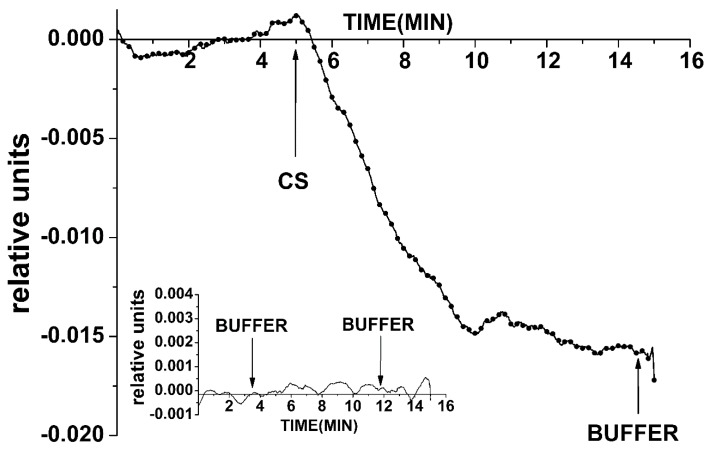
Typical sensogram obtained upon oDNA detection (cs_3) with the chip after two years of storage and subsequent intensive removal of the surface layer. Experimental conditions: 1 mM potassium phosphate buffer, *V_g_* = 55 V, *V_ds_* = 0.1 V, solution volume in the cell: 450 µL; the SOI-NW structure is sensitized with the oligonucleotide probe_3. The final concentration of cs_3 solutions in the cell is 3.3 × 10^−16^ M. The inset shows the results of control experiments. Arrows indicate the addition of the analyzed solution and washing potassium phosphate buffer into the measuring cell.

These measurements have been performed to study the inversed signal of the biosensor. After an intensive removal of the surface chip layer with five treatments of HF (see Section 2), the inversed signal from the chip disappeared upon addition of negatively charged oDNA molecules (Figure 9). Figure 9 displays a typical sensogram obtained upon the detection of oDNA at 3.3 × 10^−16^ M concentration. In our experiments, this was the lowest detectable oDNA concentration.

As one can see from this Figure, a slight “closing” of the n-type chip was observed after oDNA addition. This means that, indeed, the surface degradation properties of the chip could have a significant effect on chip conductance, even leading to the inversed signal from the chip. Thus, five-time treatment with HF provides regeneration of the chip surface, and allows the detection of oDNA at ultra-low (~10^−16^ M) concentrations.

## 4. Biospecific Detection of miRNA that Has Been Isolated from Plasma Using Three Types of SOI-NW Chips

**Figure 10:**
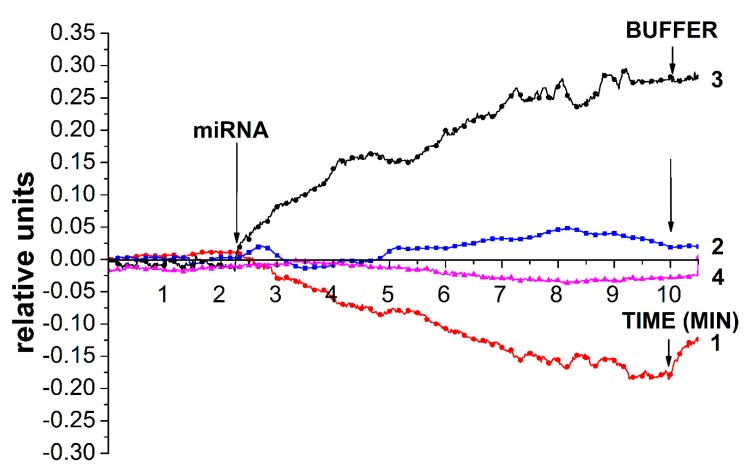
Sensograms obtained upon miRNA detection in clinical samples. Numbers for the freshly prepared chips indicate the curves obtained in the case of sample #44 from a patient with prostate cancer (curve 1, red) and in the case of sample #36 from a healthy volunteer (curve 2, blue). Chip after a two-year storage period: sample #44 of a patient with prostate cancer (curve 3, black). Chip after an intensive removal of the surface layer by five-time treatment with HF: sample #2 of a patient with prostate cancer (curve 4, purple). Experimental conditions: 1 mM potassium phosphate buffer, total solution volume in the cell: 107 μL; the SOI-NW structure with immobilized oligonucleotide probe_2. Arrows indicate the addition of the analyzed samples and washing with potassium phosphate buffer.

Below, we describe the results obtained upon the detection of miRNA that has been isolated from plasma samples of prostate cancer patients and one healthy volunteer. These results have been obtained using three types of sensor chips (see Section 2): (1) a freshly prepared one; (2) a chip stored for two years; and (3) a chip treated five times with HF to intensively remove the surface layer. Figure 10 displays the *I_ds_(t)* dependencies obtained for these chips upon analysis of the plasma of several patients. As seen in Figure 10, a decrease in the biosensor signal was observed upon using the freshly prepared chip for the analysis of a sample containing a pool of miRNAs that was isolated from plasma sample #44 (of a prostate cancer patient). Such a change in the signal has also been observed upon the detection of cs_1 oDNA (described in the Section 3.1.2) when a decrease in SOI-NW conductance has also been observed. Upon the detection of miRNA that was isolated from the plasma of a healthy volunteer (#36), the signal fluctuated near the zero line, and its level had a slight tendency to increase.

For the chip stored for two years, a significant increase in the biosensor signal (in absolute value) was noted upon addition of sample #44 into the measuring cell. This increase was similar to that obtained in the case of the freshly prepared chip, unlike the decrease in conductance in the latter case. Such a behavior—exhibited by the chip stored for two years upon addition of the sample from a cancer patient—is similar to that observed upon the addition of a complimentary oDNA (as described in Section 3.3.2). In Figure 10, the response of a chip that has been intensively treated with HF to remove its surface layer is shown in an analysis of sample #2. As one can see, the addition of this sample led to a decrease in the biosensor signal, which is similar to the case with complimentary oDNA (as described in Section 3.3.3).

## 5. Discussion

A nanowire biosensor is a very promising device for a highly sensitive bimolecular detection, since it allows for the registration of a signal from a single biomolecule adsorbed on a sensor element [18]. Protein markers such as PSA (prostate specific antigen), which are used in clinical practice, must be detected in blood with a sensitivity of 10^−17^ M in order to detect prostate cancer at an early stage [19]. We can conclude that a nanowire biosensor is suitable for this purpose, as was illustrated before. At the same time, it is known that protein markers of prostate cancer are not products of tumor cells, but are associated with the inflammatory process. We can also observe an increase in scientific publications related to the use of miRNAs for diagnosis as a result of the unique microRNA expression profiles of tumors at the very early stages of carcinogenesis [6,7,20,21].

We have investigated the possibility of biospecific detection of model DNA oligonucleotides complimentary to a probe for PCa-associated miRNAs. It was shown that oDNAs, which are synthetic analogues of PCa-associated miRNAs, can be effectively detected in a buffer solution with the use of a nanowire biosensor with high sensitivity (~3.3 × 10^−16^ M) in real time without the use of additional labels.

One of the most important characteristics of chips for the SOI-NW biosensor is their change in properties during long-term storage. We have studied the influence of chip storage times on their properties, and it has been demonstrated that, in the case of a one-year storage of SOI chips, no significant changes in the properties of the chips could be observed. At the same time, when the chips were stored for two years, one can see an interesting change in the chips’ properties that results in an inversed signal upon the detection of oDNA molecules. The reason for the change in characteristics during long-term storage (i.e., two years) may be the influence of atmospheric oxygen on natural oxides, which are formed on the outer surfaces of nanowire sensor elements. Bohling has shown that the properties of an oxide can change dramatically upon contact with the environment [22], and the mechanism causing the difference in response from the freshly prepared chip with that from the chip stored for two years could be explained in the following way. On the surface of a freshly prepared chip, the molecules of covalently immobilized oDNA probes can have an arcuate shape (reaching to the surface of the chip), as the negatively charged bases are attracted to the positively charged nanowire surface, whose positive net charge is caused by application of positive voltage to the gate.

Over time (after two years of storage in our present study), islands with excessive negative charges are formed on the chip’s surface. The accumulation of a negative charge on the chip’s surface can be caused by the following factors: (1) the presence of negative ions in the environment; (2) a negatively charged layer formed during oxidation; or (3) the presence of a negative layer in silicon dioxide due to excessive interstitial oxygen. Accordingly, the surface of a chip stored for two years bears an additional (with respect to the charge of the freshly prepared chip) negative charge due to the negative defects, and this charge compensates the positive charge of the gate, which causes the oDNA probes—immobilized on the surface of the old chip—to be less attracted to the nanowire surface, as compared with a freshly prepared chip.

In the case of a freshly prepared chip, upon the addition of oDNA into the cell the oDNA base sequence hybridizes to the complementary base sequence of the immobilized oDNA probes, while the positively charged amino group NH2+ of the oDNA moves up from the substrate due to the repulsion caused by the positive potential of the gate. Taking into account that the oDNA sequence contains 84 to 110 base pairs, its linear length makes up 29 to 37 nm, and these groups can leave the zone of Debye length. Because of this, negatively charged bases of the oDNA, hybridizing on the surface, cause a decrease in the chip’s conductance. In the case of the chip stored for two years, the oDNA base sequence hybridizes with the sequence of complementary bases of immobilized oDNA probes, but the excess negative charge of the chip surface, which neutralizes the positive charge on the nanowire surface, acts in such a way that the positively charged NH^2+^ group of the oDNA is attracted very close to the surface. This is what leads to the “opening” of the chip transistor and, as a result, to the observed inversion mode.

In the case of plasma containing a pool of microRNAs, these microRNAs most likely present in the form of complexes with positively charged proteins, which can cause an excessive positive charge on the microRNA-protein structure. The differently directed response to hybridization of plasma microRNAs on the surface of new and old chips will be similar to that given above for the case of experiments with synthetic oDNA structures.

We have also demonstrated that the use of a procedure for intensive removal of the surface layer from the chip can allow one to convert the inversed signal from the chip to the expected direct one, as tested upon the addition of negatively charged oDNA. An interesting effect of the chip’s inverted conductance upon modification of its surface layer during long-term storage is that it is the surface (not the whole cross-section of the conductor) that forms the response to the biosensor on nucleic acids such as oDNA and miRNA.

In our study, the capability of SOI-NW chips to detect miRNAs associated with prostate cancer in plasma samples from patients has been demonstrated. In the case of a freshly prepared n-type chip, an anticipated decrease in conductance was observed upon addition of a sample containing negatively charged miRNA. Inverse sensitivity of the chip after two years of storage was observed not only in the case of oDNA, but also in the case of corresponding miRNA. After intensive removal of the oxide surface layer of the chip that was stored for two years using five treatments with HF, chip conductivity returned from the inverse to the direct mode of detection for both the oDNA and miRNA in the blood plasma of a cancer patient.

The results obtained should be taken into account in the development of highly sensitive nanowire-based diagnostic systems for the revelation of (prostate) cancer-associated microRNAs in human plasma.

## 6. Conclusions

SOI-NW biosensor chips were manufactured with the use of CMOS-compatible technology by gas phase reduction and lithography. We were able to demonstrate the ability of such a SOI-NW biosensor in order to record oDNA with high sensitivity at a level of 3.3 × 10^−16^ M on a real-time basis without the use of labels. SOI-NW biosensor chips can be used for one year. After two years of storage, the chips “become old”, which leads to the appearance of an inversed signal during the registration of negatively infected oDNAs. The discovered effect of inverting the chip’s conductance upon modification of its surface layer indicates its significant contribution to providing the sensitivity of the chip toward nucleic acids. The possible use of SOI-NW biosensor chips for the revelation of microRNAs in plasma has been demonstrated. The results obtained herein are of importance for the development of nanowire-based diagnostic systems for the highly sensitive detection of cancer-associated microRNAs in human plasma.

## Figures and Tables

**Table 1:** DNA oligonucleotide probe sequences.

DNA Oligonucleotide Name	DNA Oligonucleotide Sequence	Type of microRNA Related
probe 1	(NH_2_)T_10_TCGTGGATCTGTCTCTGCTCTGTTTATGGCCCTTCGGTAATTCACTGACTGAGACTGTTCACAGTGATTTCTACCAGTGCCATACACAGAACAGGAGTCACACTCTGCGG	hsa-mir-198 [15]
probe 3	(NH_2_)T_10_GCAGCGGATGGACGGTTTTACCAGACAGTATTAGACAGAGGGCCAGGTCTAACCATGTTCTGGTAAGACGCCCATGGCCGGCG	hsa-mir-429 [15]

**Table 2:** Sequences of oDNA and miRNA.

DNA Oligonucleotide Name	DNA Oligonucleotide Sequence	Type of microRNA Related
*cs_1*	CCGCAGAGTGTGACTCCTGTTCTGTGTATGGCACTGGTAGAATTCACTGTGAACAGTCTCAGTCAGTGAATTACCGAAGGGCCATAAACAGAGCAGAGACAGATCCACGA	microRNA 198 [15]
*cs_3*	CGCCGGCCGATGGGCGTCTTACCAGACATGGTTAGACCTGGCCTCTGTCTAATACTGTCTGGTAAAACCGTCCATCCGCTGC	microRNA 429 [15]

**Table 3:** Clinical and morphological characteristics of prostate cancer (PCa) patients and one healthy volunteer.

Plasma Sample #	Age	Pathology	Degree of Tumor Differentiation (G)	Stage According to TNM System
No. 2	67	prostate carcinoma	G1-G2	T2cN0M0
No. 44	68	acinar prostate adenocarcinoma	G1-G2	T1cN0M0
No. 36	62	healthy volunteer	-	-
